# Universal Unitarity Triangle 2016 and the tension between $\Delta M_{s,d}$ and $\varepsilon _K$ in CMFV models

**DOI:** 10.1140/epjc/s10052-016-4044-6

**Published:** 2016-04-11

**Authors:** Monika Blanke, Andrzej J. Buras

**Affiliations:** 1 Institut fur Kernphysik, Karlsruhe Institute of Technology, Hermann-von-Helmholtz-Platz 1, 76344 Eggenstein-Leopoldshafen, Germany; 2 Institut fur Theoretische Teilchenphysik, Karlsruhe Institute of Technology, Engesserstraße 7, 76128 Karlsruhe, Germany; 3 TUM-IAS, Lichtenbergstr. 2a, 85748 Garching, Germany; 4 Physik Department, TUM, 85748 Garching, Germany

## Abstract

Motivated by the recently improved results from the Fermilab Lattice and MILC Collaborations on the hadronic matrix elements entering $\Delta M_{s,d}$ in $B_{s,d}^0$–$\bar{B}_{s,d}^0$ mixing, we determine the universal unitarity triangle (UUT) in models with constrained minimal flavour violation (CMFV). Of particular importance are the very precise determinations of the ratio $|V_{ub}|/|V_{cb}|=0.0864\pm 0.0025$ and of the angle $\gamma =(62.7\pm 2.1)^\circ$. They follow in this framework from the experimental values of $\Delta M_{d}/\Delta M_s$ and of the CP-asymmetry $S_{\psi K_S}$. As in CMFV models the new contributions to meson mixings can be described by a single flavour-universal variable *S*(*v*), we next determine the CKM matrix elements $|V_{ts}|$, $|V_{td}|$, $|V_{cb}|$ and $|V_{ub}|$ as functions of *S*(*v*) using the experimental value of $\Delta M_s$ as input. The lower bound on *S*(*v*) in these models, derived by us in 2006, implies then *upper* bounds on these four CKM elements and on the CP-violating parameter $\varepsilon _K$, which turns out to be significantly below its experimental value. This strategy avoids the use of tree-level determinations of $|V_{ub}|$ and $|V_{cb}|$, which are presently subject to considerable uncertainties. On the other hand, if $\varepsilon _K$ is used instead of $\Delta M_s$ as input, $\Delta M_{s,d}$ are found to be significantly above the data. In this manner we point out that the new lattice data have significantly sharpened the tension between $\Delta M_{s,d}$ and $\varepsilon _K$ within the CMFV framework. This implies the presence of new physics contributions beyond this framework that are responsible for the breakdown of the flavour universality of the function *S*(*v*). We also present the implications of these results for $K^+\rightarrow \pi ^+\nu \bar{\nu }$, $K_{L}\rightarrow \pi ^0\nu \bar{\nu }$ and $B_{s,d}\rightarrow \mu ^+\mu ^-$ within the Standard Model.

## Introduction

Already for decades the $\Delta F=2$ transitions in the down-quark sector, that is $B^0_{s,d}$–$\bar{B}^0_{s,d}$ and $K^0$–$\bar{K}^0$ mixings, have been vital in constraining the Standard Model (SM) and in the search for new physics (NP) [1, 2]. However, theoretical uncertainties related to the hadronic matrix elements entering these transitions and their large sensitivity to the CKM parameters so far precluded clear cut conclusions about the presence of new physics (NP).

The five observables of interest are

1 $$\begin{aligned} \Delta M_s,\quad \Delta M_d, \quad S_{\psi K_S},\quad S_{\psi \phi }, \quad \varepsilon _K \end{aligned}$$

with $\Delta M_{s,d}$ being the mass differences in $B^0_{s,d}$–$\bar{B}^0_{s,d}$ mixings and $S_{\psi K_S}$ and $S_{\psi \phi }$ the corresponding mixing induced CP-asymmetries. $\varepsilon _K$ describes the size of the indirect CP-violation in $K^0$–$\bar{K}^0$ mixing. $\Delta M_{s,d}$ and $\varepsilon _K$ are already known with impressive precision. The asymmetries $S_{\psi K_S}$ and $S_{\psi \phi }$ are less precisely measured but have the advantage of being subject to only very small hadronic uncertainties. We do not include $\Delta M_K$ in (1) as it is subject to much larger theoretical uncertainties than the five observables in question.

The hadronic uncertainties in $\Delta M_{s,d}$ and $\varepsilon _K$ within the SM and CMFV models reside within a good approximation in the parameters

2 $$\begin{aligned} F_{B_s}\sqrt{\hat{B}_{B_s}},\quad F_{B_d} \sqrt{\hat{B}_{B_d}}, \quad \hat{B}_K. \end{aligned}$$

Fortunately, during the last years these uncertainties decreased significantly. In particular, concerning $F_{B_s}\sqrt{\hat{B}_{B_s}}$ and $F_{B_d} \sqrt{\hat{B}_{B_d}}$, an impressive progress has recently been made by the Fermilab Lattice and MILC Collaborations (Fermilab-MILC) that find [3]

3 $$\begin{aligned} F_{B_s}\sqrt{\hat{B}_{B_s}}&=(276.0\pm 8.5)\, \mathrm{MeV},\nonumber \\ F_{B_d} \sqrt{\hat{B}_{B_d}}&= (229.4\pm 9.3)\, \mathrm{MeV}, \end{aligned}$$

with uncertainties of 3 % and 4 %, respectively. An even higher precision is achieved for the ratio

4 $$\begin{aligned} \xi =\frac{F_{B_s}\sqrt{\hat{B}_{B_s}}}{F_{B_d}\sqrt{\hat{B}_{B_d}}}=1.203\pm 0.019. \end{aligned}$$

This value is significantly lower than the central value 1.27 in the previous lattice estimates [4] and its reduced uncertainty by a factor of three plays an important role in our analysis. The ETM Collaboration has also presented results for matrix elements of all five operators entering $B_{d,s}$–$\bar{B}_{d,s}$ mixing [5]. This work, however, only employs two flavours of sea quarks and does not estimate the uncertainty from quenching the strange quark. The ETM and Fermilab-MILC results for matrix elements differ by $\sim 5.5$ %, or $\sim 1\sigma$, which could arise from the omitted strange sea. We think it is safer to avoid this issue and use only the Fermilab-MILC results with $N_f=2+1$. However, we note that the result for $\xi$ obtained by the ETM Collaboration supports a rather low value of $\gamma$ from the Universal Unitarity Triangle (UUT). An extensive list of references to other lattice determinations of these parameters can be found in [3].

Lattice QCD also made impressive progress in the determination of the parameter $\hat{B}_K$, which enters the evaluation of $\varepsilon _K$ [6–11]. The most recent preliminary world average from FLAG reads $\hat{B}_K=0.7627 (97)$ [12], very close to its large *N* value $\hat{B}_K=0.75$ [13, 14]. Moreover, the analyses in [15, 16] show that $\hat{B}_K$ cannot be larger than 0.75 but must be close to it. Taking the present results and precision of lattice QCD into account it is then a good approximation to set $\hat{B}_K=0.750 \pm 0.015$. In the evaluation of $\varepsilon _K$ we also take into account long distance contributions parametrised by $\kappa _\varepsilon = 0.94\pm 0.02$ [17]. Note that at present the theoretical uncertainty in $\varepsilon _K$ is dominated by the parameter $\eta _{cc} = 1.87 \pm 0.76$ [18] summarising NLO and NNLO QCD corrections to the charm quark contribution. We take these uncertainties into account.

With $|V_{us}|$ determined already very precisely, the main uncertainties in the CKM parameters reside in

5 $$\begin{aligned} |V_{cb}|, \quad |V_{ub}|, \quad \gamma , \end{aligned}$$

with $\gamma$ being one of the angles of the unitarity triangle (UT). These three parameters can be determined from tree-level decays that are subject to only very small NP contributions. However, the tensions between inclusive and exclusive determinations of $|V_{ub}|$ and to a lesser extent of $|V_{cb}|$ do not yet allow for clear cut conclusions on their values. Moreover, the current world average of direct measurements of $\gamma$ is not precise [19]

6 $$\begin{aligned} \gamma = \left( 73.2^{+6.3}_{-7.0}\right) ^\circ . \end{aligned}$$

This is consistent with $\gamma$ from the U-spin analysis of $B_s\rightarrow K^+K^-$ and $B_d\rightarrow \pi ^+\pi ^-$ decays ($\gamma =(68.2\pm 7.1)^\circ$) [20]. The U-spin analysis by LHCb [21], on the other hand, finds a lower value $\gamma = ( 63.5^{+7.2}_{-6.7})^\circ$ in good agreement with the result from the UUT analysis in (25).

The present uncertainties in $|V_{ub}|/|V_{cb}|$ and $\gamma$ from tree-level decays preclude then a precise determination of the so-called *reference unitarity triangle* (RUT) [22] which is expected to be practically independent of the presence of NP. In addition the uncertainty in $|V_{cb}|$ prevents precise predictions for $\varepsilon _K$ and $\Delta M_{s,d}$ in the SM. However, in the SM and more generally models with constrained minimal flavour violation (CMFV) [23–25] it is possible to construct the so-called *Universal Unitarity Triangle* (UUT) [23] for which the knowledge of $|V_{ub}|/|V_{cb}|$ and $\gamma$ is not required. The UUT can be constructed from

7 $$\begin{aligned} \frac{\Delta M_d}{\Delta M_s}, \quad S_{\psi K_S} \end{aligned}$$

and this in turn allows one to determine $|V_{ub}|/|V_{cb}|$ and $\gamma$.

The important virtue of this determination is its universality within CMFV models. In the case of $\Delta F=2$ transitions in the down-quark sector various CMFV models can only be distinguished by the value of a single flavour-universal real one-loop function, the box diagram function *S*(*v*), with *v* collectively denoting the parameters of a given CMFV model. This function enters universally $\varepsilon _K$, $\Delta M_s$ and $\Delta M_d$ and cancels out in the ratio in (7). Therefore the resulting UUT is the same in all CMFV models. Moreover, it can be shown that in these models *S*(*v*) is bounded from below by its SM value [26]

8 $$\begin{aligned} S(v)\ge S_0(x_t)= 2.32 \end{aligned}$$

with $S_0(x_t)$ given in (11).

The recent results in (3) and (4) have a profound impact on the determination of the UUT. The UUT can be determined very precisely from the measured values of $\Delta M_d/\Delta M_s$ and $S_{\psi K_S}$. This in turn implies a precise knowledge of the ratio $|V_{ub}|/|V_{cb}|$ and the angle $\gamma$, both to be compared with their tree-level determinations. Also the side $R_t$ of the UUT can be determined precisely in view of the result for $\xi$ in (4).

In order to complete the determination of the full CKM matrix without the use of any tree-level determinations, except for $|V_{us}|$, we will use two strategies:

- ${\varvec{S}_1}$: ${{\varvec{\Delta }}M_s}$ **strategy** in which the experimental value of $\Delta M_s$ is used to determine $|V_{cb}|$ as a function of *S*(*v*), and $\varepsilon _K$ is then a derived quantity.
- ${\varvec{S}_2}$: ${\varvec{\varepsilon }_K}$ **strategy** in which the experimental value of $\varepsilon _K$ is used, while $\Delta M_{s}$ is then a derived quantity and $\Delta M_d$ follows from the determined UUT.

Both strategies use the determination of the UUT by means of (7) and allows to determine the whole CKM matrix, in particular $|V_{ts}|$, $|V_{td}|$, $|V_{ub}|$ and $|V_{cb}|$ as functions of *S*(*v*). Yet their outcome is very different, which signals the tension between $\Delta M_{s,d}$ and $\varepsilon _K$ in this framework. As we will demonstrate below, this tension, known already from previous studies [27, 28], has been sharpened significantly through the results in (3) and (4). Using these two strategies separately allows one to exhibit this tension transparently. Indeed we have the following:

- The lower bound in (8) implies in $S_1$ *upper bounds* on $|V_{ts}|$, $|V_{td}|$, $|V_{ub}|$ and $|V_{cb}|$ which are saturated in the SM, and in turn it allows to derive an *upper bound* on $\varepsilon _K$ in CMFV models that is saturated in the SM but turns out to be significantly *below* the data.
- The lower bound in (8) implies in $S_2$ also *upper bounds* on $|V_{ts}|$, $|V_{td}|$, $|V_{ub}|$ and $|V_{cb}|$ which are saturated in the SM. However the *S*(*v*) dependence of these elements determined in this manner differs from the one obtained in $S_1$, which in turn allows to derive *lower bounds* on $\Delta M_{s,d}$ in CMFV models that are reached in the SM but turn out to be significantly *above* the data.

It has been known since 2008 that the SM experiences some tension in the correlation between $S_{\psi K_S}$ and $\varepsilon _K$ [29–33]. It should be emphasised that in CMFV models only the version of this tension in [30], i. e. NP in $\varepsilon _K$, is possible as in these models there are no new CP-violating phases. Therefore $S_{\psi K_S}$ has to be used to determine the sole phase in these models, the angle $\beta$ in the UT, or equivalently the CKM phase, through the unitarity of the CKM matrix. The resulting low value of $\varepsilon _K$ can be naturally raised in CMFV models by enhancing the value of *S*(*v*) and/or increasing the value of $|V_{cb}|$. However, as pointed out in [27, 28], this spoils the agreement of the SM with the data on $\Delta M_{s,d}$, signalling the tension between $\Delta M_{s,d}$ and $\varepsilon _K$ in CMFV models. The 2013 analysis of this tension in [34] found that the situation of CMFV with respect to $\Delta F=2$ transitions would improve if more precise results for $F_{B_s}\sqrt{\hat{B}_{B_s}}$ and $F_{B_d} \sqrt{\hat{B}_{B_d}}$ turned out to be lower than the values known in the spring of 2013. The recent results from [3] in (3) show the opposite. Both $F_{B_s}\sqrt{\hat{B}_{B_s}}$ and $F_{B_d} \sqrt{\hat{B}_{B_d}}$ increased. Moreover the more precise and significantly smaller value of $\xi$ enlarges the tension in question.

In view of the new lattice results, in this paper we take another look at CMFV models. Having more precise values for $F_{B_s}\sqrt{\hat{B}_{B_s}}$, $F_{B_d} \sqrt{\hat{B}_{B_d}}$ and $\xi$ than in 2013, our strategy outlined above differs from the one in [34]. In particular we take $\gamma$ to be a derived quantity and not an input as done in the latter paper. Moreover, we will be able to reach much firmer conclusions than it was possible in 2013. In particular, in contrast to [34] and also to [3] at no place in our paper tree-level determinations of $|V_{ub}|$, $|V_{cb}|$ and $\gamma$ are used. However, we compare our results with them.

It should be mentioned that Fermilab-MILC identified a significant tension between their results for the $B^0_{s,d}-\bar{B}^0_{s,d}$ mass differences and the tree-level determination of the CKM matrix within the SM. Complementary to their findings, we identify a significant tension within $\Delta F = 2$ processes, that is between $\varepsilon _K$ and $\Delta M_{s,d}$ in the whole class of CMFV models. Moreover, we determine very precisely the UUT, in particular the angle $\gamma$ in this triangle and the ratio $|V_{ub}|/|V_{cb}|$, both valid also in the SM.

Our paper is organised as follows. In Sect. 2 we determine first the UUT as outlined above, that in 2016 is significantly better known than in 2006 [25] and in particular in 2000, when the UUT was first suggested [23]. Subsequently we execute the strategies $S_1$ and $S_2$ defined above. The values of $|V_{ts}|$, $|V_{td}|$, $|V_{cb}|$ and $|V_{ub}|$, resulting from these two strategies, differ significantly from each other which is the consequence of the tension between $\varepsilon _K$ and $\Delta M_{s,d}$ in question. In Sect. 3 we present the implications of these results for $K_{L}\rightarrow \pi ^0\nu \bar{\nu }$, $K^+\rightarrow \pi ^+\nu \bar{\nu }$ and $B_{s,d}\rightarrow \mu ^+\mu ^-$ within the SM, obtaining again rather different results in $S_1$ and $S_2$. In Sect. 4 we briefly discuss how the $U(2)^3$ models match the new lattice data and comment briefly on other models. We conclude in Sect. 5.

## Deriving the UUT and the CKM

### Determination of the UUT

We begin with the determination of the UUT. For the mass differences in the $B^0_{s,d}-\bar{B}^0_{s,d}$ systems we have the very accurate expressions

9 $$\begin{aligned} \Delta M_{s}= & {} 17.757/\mathrm{ps}\cdot \left[ \frac{\sqrt{\hat{B}_{B_s}}F_{B_s}}{276.0\, \mathrm{MeV}}\right] ^2 \left[ \frac{S(v)}{2.322}\right] \left[ \frac{|V_{ts}|}{0.0389} \right] ^2\nonumber \\&\times \left[ \frac{\eta _B}{0.5521}\right] ,\end{aligned}$$

10 $$\begin{aligned} \Delta M_d= & {} 0.5055/\mathrm{ps}\cdot \left[ \frac{\sqrt{\hat{B}_{B_d}}F_{B_d}}{229.4\, \mathrm{MeV}}\right] ^2 \left[ \frac{S(v)}{2.322}\right] \nonumber \\&\times \left[ \frac{|V_{td}|}{7.95\cdot 10^{-3}} \right] ^2 \left[ \frac{\eta _B}{0.5521}\right] . \end{aligned}$$

The value 2.322 in the normalisation of *S*(*v*) is its SM value for $m_t(m_t)=163.5\, \mathrm{GeV}$ obtained from

11 $$\begin{aligned} S_0(x_t)= & {} \frac{4x_t - 11 x_t^2 + x_t^3}{4(1-x_t)^2}-\frac{3 x_t^2\log x_t}{2 (1-x_t)^3}\nonumber \\= & {} 2.322 \left[ \frac{\overline{m}_\mathrm{t}(m_\mathrm{t})}{163.5\, \mathrm{GeV}}\right] ^{1.52}, \end{aligned}$$

and $\eta _B$ is the perturbative QCD correction [35]. Our input parameters, equal to the ones used in [3], are collected in Table 1.

**Table 1:** Values of the experimental and theoretical quantities used as input parameters. For future updates see PDG [36] and HFAG [37]

$m_{B_s} = 5366.8 (2)\, \mathrm{MeV}$ [36]	$m_{B_d}=5279.58 (17)\, \mathrm{MeV}$ [36]
$\Delta M_s = 17.757 (21) \,\text {ps}^{-1}$ [37]	$\Delta M_d = 0.5055 (20) \,\text {ps}^{-1}$ [37]
$S_{\psi K_S}= 0.691 (17)$ [37]	$S_{\psi \phi }= 0.015 (35)$ [37]
$|V_{us}|=0.2253 (8)$ [36]	$|\varepsilon _K|= 2.228 (11)\cdot 10^{-3}$ [36]
$F_{B_s}$ = $226.0 (22)\, \mathrm{MeV}$ [38]	$F_{B_d}$ = $188 (4)\, \mathrm{MeV}$ [39]
$m_t(m_t)=163.53 (85)\, \mathrm{GeV}$	$S_0(x_t)=2.322 (18)$
$\eta _{cc}=1.87 (76)$ [18]	$\eta _{ct}= 0.496 (47)$ [40]
$\eta _{tt}=0.5765 (65)$ [35]	$\eta _B=0.55 (1)$ [35, 41]
$\tau _{B_s}= 1.510 (5)\,\text {ps}$ [37]	$\Delta \Gamma _s/\Gamma _s=0.124 (9)$ [37]
$\tau _{B_d}= 1.520 (4)\,\text {ps}$ [37]	$\kappa _\varepsilon = 0.94 (2)$ [17]

From (9) and (10) we find using (4)

12 $$\begin{aligned} \frac{|V_{td}|}{|V_{ts}|}=\xi \sqrt{\frac{m_{B_s}}{m_{B_d}}}\sqrt{\frac{\Delta M_d}{\Delta M_s}}= 0.2046\pm 0.0033, \end{aligned}$$

which perfectly agrees with [3]. The tree-level determination of this ratio, quoted in the latter paper and obtained from CKMfitter [42], reads

13 $$\begin{aligned} \frac{|V_{td}|_\text {tree}}{|V_{ts}|_\text {tree}}=0.2180\pm 0.0031. \end{aligned}$$

It is significantly higher than the value in (12). It should be emphasised that the values of $|V_{cb}|$ and $|V_{ub}|$ to a very good approximation do not enter this ratio. Therefore this discrepancy is not a consequence of the tree-level determinations of $|V_{cb}|$ and $|V_{ub}|$. As we will demonstrate below it is the consequence of the value of the angle $\gamma$, which due to the small value of $\xi$ found in [3] turns out to be significantly smaller than its tree-level value in (6).

**Fig. 1:**
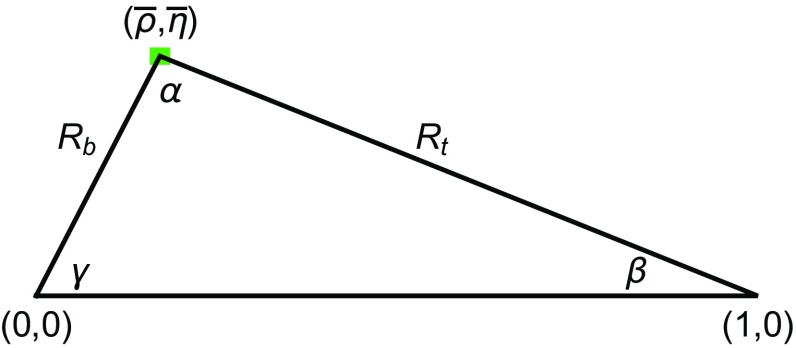
Universal Unitarity Triangle 2016. The *green square* at the apex of the UUT shows that the uncertainties in this triangle are impressively small

Now,

14 $$\begin{aligned} |V_{td}|=|V_{us}||V_{cb}|R_t,\quad |V_{ts}|=\eta _R|V_{cb}|~ \end{aligned}$$

with $R_t$ being one of the sides of the unitarity triangle (see Fig. 1) and

15 $$\begin{aligned} \eta _R= & {} 1 -|V_{us}|\xi \sqrt{\frac{\Delta M_d}{\Delta M_s}}\sqrt{\frac{m_{B_s}}{m_{B_d}}}\cos \beta +\frac{\lambda ^2}{2}+\mathcal{O}(\lambda ^4)\nonumber \\= & {} 0.9826, \end{aligned}$$

where we have used

16 $$\begin{aligned} \beta =(21.85\pm 0.67)^\circ \, \end{aligned}$$

obtained from

17 $$\begin{aligned} S_{\psi K_S}=\sin 2\beta =0.691 \pm 0.017. \end{aligned}$$

**Fig. 2:**
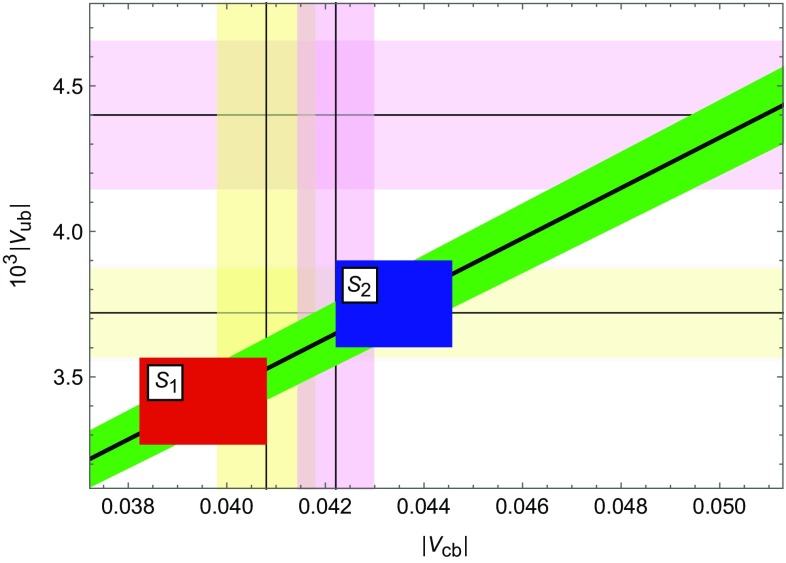
$|V_{ub}|$ versus $|V_{cb}|$ in CMFV (*green*) compared with the tree-level exclusive (*yellow*) and inclusive (*violet*) determinations. The *squares* are our results in $S_1$ (*red*) and $S_2$ (*blue*)

Thus using (12) and (14) we determine very precisely

18 $$\begin{aligned} R_t= 0.741 \, \xi = 0.893\pm 0.013. \end{aligned}$$

Having determined $\beta$ and $R_t$ we can construct the UUT shown in Fig. 1, from which we find

19 $$\begin{aligned} {\bar{\rho }}=0.172\pm 0.013,\quad {\bar{\eta }}= 0.332\pm 0.011. \end{aligned}$$

We observe that the UUT in Fig. 1 differs significantly from the UT obtained in global fits [42, 43], with the latter exhibiting smaller ${\bar{\rho }}$ and larger ${\bar{\eta }}$ values.

Subsequently, using the relation

20 $$\begin{aligned} R_b=\left( 1-\frac{\lambda ^2}{2}\right) \frac{1}{\lambda } \left| \frac{V_{ub}}{V_{cb}} \right| = \sqrt{1+R_t^2-2 R_t\cos \beta } \end{aligned}$$

allows for a very precise determination of the ratio

21 $$\begin{aligned} \frac{|V_{ub}|}{|V_{cb}|}= 0.0864\pm 0.0025. \end{aligned}$$

This implies, as shown in Fig. 2, a strict correlation between $|V_{ub}|$ and $|V_{cb}|$, which can be compared with the tree-level determinations of both CKM elements, also shown in this plot. The exclusive determinations [3, 44–46] give

22 $$\begin{aligned} |V_{cb}|_\text {excl}= & {} (40.8\pm 1.0)\cdot 10^{-3},\nonumber \\ |V_{ub}|_\text {excl}= & {} (3.72\pm 0.16)\cdot 10^{-3} \end{aligned}$$

and the inclusive ones [47]

23 $$\begin{aligned} |V_{cb}|_\text {incl}= & {} (42.21\pm 0.78)\cdot 10^{-3},\nonumber \\ |V_{ub}|_\text {incl}= & {} (4.40\pm 0.25)\cdot 10^{-3}. \end{aligned}$$

We note that after the recent Belle data on $B\rightarrow D\ell \nu _l$ [46], the exclusive and inclusive values of $|V_{cb}|$ do not differ by much, while in the case of $|V_{ub}|$ there is a significant difference. Moreover, the recent result on $|V_{ub}|$ from LHCb with $|V_{ub}|=3.27(23)\cdot 10^{-3}$ [48] favours its lower value in (22).

**Table 2:** Upper bounds on CKM elements in units of $10^{-3}$ and of $\lambda _t$ in units of $10^{-4}$ obtained using strategies $S_1$ and $S_2$ as explained in the text. We set $S(v)=S_0(x_t)$

$S_i$	$|V_{ts}|$	$|V_{td}|$	$|V_{cb}|$	$|V_{ub}|$	$\mathrm{Im}\lambda _t$	$\mathrm{Re}\lambda _t$
$S_1$	$38.9\,(13)$	$7.95\,(29)$	$39.5\,(1.3)$	$3.41\,(15)$	$1.20\,(8)$	$-2.85\,(19)$
$S_2$	$42.7\,(12)$	$8.74\,(27)$	$43.4\,(1.2)$	$3.75\,(15)$	$1.44\,(8)$	$-3.44\,(19)$

We observe that within the CMFV framework only special combinations of these two CKM elements are allowed. The *red* and *blue* squares represent the ranges obtained in the strategies $S_1$ and $S_2$, respectively, as explained below and summarised in Table 2. We observe significant tensions both between the results in $S_1$ and $S_2$ and also between them and the inclusive tree-level determination of $|V_{ub}|$. On the other hand, the exclusive determination of $|V_{ub}|$ accompanied by the inclusive one for $|V_{cb}|$ gives $|V_{ub}|/|V_{cb}|=0.0881\pm 0.0041$, very close to the result in (21). However, the separate values of $|V_{ub}|$ and $|V_{cb}|$ in (22) and (23) used to obtain this result are not compatible with our findings in $S_1$, implying problems with $\Delta M_{s,d}$ as we will see below.

Returning to the issue of the origin of the difference between (12) and (13), the new lattice results [3] have important implications on the angle $\gamma$ in the UUT that can be determined by means of

24 $$\begin{aligned} \cot \gamma =\frac{1-R_t\cos \beta }{R_t\sin \beta }. \end{aligned}$$

**Fig. 3:**
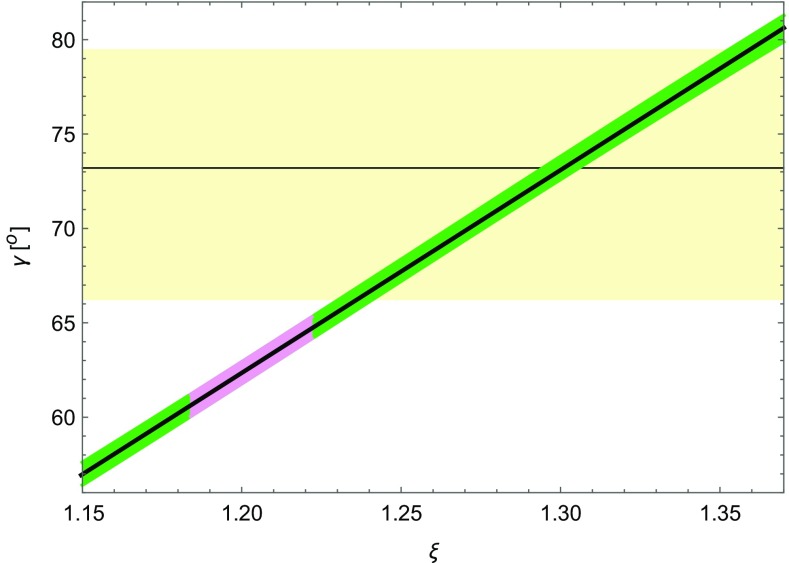
$\gamma$ versus $\xi$ for $S_{\psi K_S}=0.691\pm 0.017$. The *violet range* corresponds to the new lattice determination of $\xi$ in (4), and the *yellow range* displays the tree-level determination of $\gamma$ (6)

With the very precise value of $\xi$ and consequently $R_t$ we can precisely determine the angle $\gamma$ independently of the values of *S*(*v*), $|V_{ub}|$ and $|V_{cb}|$. In Fig. 3 we show $\gamma$ as a function of $\xi$ from which we extract

25 $$\begin{aligned} \gamma =(62.7\pm 2.1)^\circ , \end{aligned}$$

below its central value from tree-level decays in (6), and with an uncertainty that is by a factor of three smaller. We will use this value in what follows. We note that the uncertainty due to $S_{\psi K_S}$ is very small. In order to appreciate this result one can read off the plot in Fig. 3 that the old range of $\xi =1.27\pm 0.06$ corresponds to $\gamma =(70\pm 6)^\circ$.

Finally, from (16) and (25) we determine the angle $\alpha$ in the unitarity triangle

26 $$\begin{aligned} \alpha = (95.5\pm 2.2)^\circ . \end{aligned}$$

It should be emphasised that the results in (16), (18), (21), (25) and (26) are independent of *S*(*v*) and therefore valid for all CMFV models.

### $S_1$: upper bounds on $|V_{ts}|$, $|V_{td}|$, $|V_{cb}|$, $|V_{ub}|$ and $\varepsilon _K$

Returning to (9) and (10), we note that the overall factors on the r.h.s. equal the central experimental values of $\Delta M_s$ and $\Delta M_d$, respectively. We can therefore read off from these formulae the central values of $|V_{ts}|$ and $|V_{td}|$ corresponding to the lattice results in (3). Including the uncertainties in the latter formula and taking into account the inequality (8) we find the *maximal* values of $|V_{ts}|$ and $|V_{td}|$ in the CMFV models that are consistent with the data on $\Delta M_s$ and $\Delta M_d$

27 $$\begin{aligned} |V_{ts}|_\text {max}&=(38.9 \pm 1.3)\cdot 10^{-3}, \nonumber \\ |V_{td}|_\text {max}&= (7.95\pm 0.29)\cdot 10^{-3}. \end{aligned}$$

It should be noted that

28 $$\begin{aligned} |V_{ts}|&=38.9\cdot 10^{-3}\,\sqrt{\frac{2.322}{S(v)}}, \nonumber \\ |V_{td}|&= 7.95\cdot 10^{-3}\,\sqrt{\frac{2.322}{S(v)}}, \end{aligned}$$

where we suppressed the errors given in (27). Thus the bounds in (27) are saturated in the SM. The results within the SM are in excellent agreement with those obtained in [3]. Yet, here we also stress that these are upper bounds in CMFV models. Therefore, the tension between the values of these CKM elements extracted from $\Delta M_{s,d}$ and their tree-level determinations found in [3] within the SM is larger in any other CMFV model. Interestingly the values of $|V_{td}|$ and $|V_{td}|/|V_{ts}|$ extracted from the rare semileptonic decays $B\rightarrow \pi \mu ^+\mu ^-$ and $B\rightarrow K\mu ^+\mu ^-$ agree with the ones in (28) and (12), respectively [49]:

29 $$\begin{aligned}&\frac{|V_{td}|}{|V_{ts}|}=0.201(20), \nonumber \\&|V_{ts}|=35.7(1.5)\cdot 10^{-3}\quad |V_{td}|=7.45(69)\cdot 10^{-3}. \end{aligned}$$

For $|V_{ts}|$, the values are found to be even smaller than in (28). However, this determination of CKM parameters still suffers from large uncertainties. We refer to [3] for a more detailed comparison of rare semileptonic *B*-decays with $B_{s,d}$ mixing results and the relevant references.

With the knowledge of $|V_{us}|$, $|V_{ts}|$, $|V_{td}|$ and $\beta$ we can determine $|V_{ub}|$ and $|V_{cb}|$ as functions of *S*(*v*) so that they can directly be compared with their determinations from semileptonic decays summarised in (22) and (23). We find

30 $$\begin{aligned} |V_{cb}|= & {} (39.5\pm 1.3)\cdot 10^{-3}\,\sqrt{\frac{2.322}{S(v)}}, \nonumber \\ |V_{ub}|= & {} (3.41\pm 0.15)\cdot 10^{-3}\,\sqrt{\frac{2.322}{S(v)}}. \end{aligned}$$

**Fig. 4:**
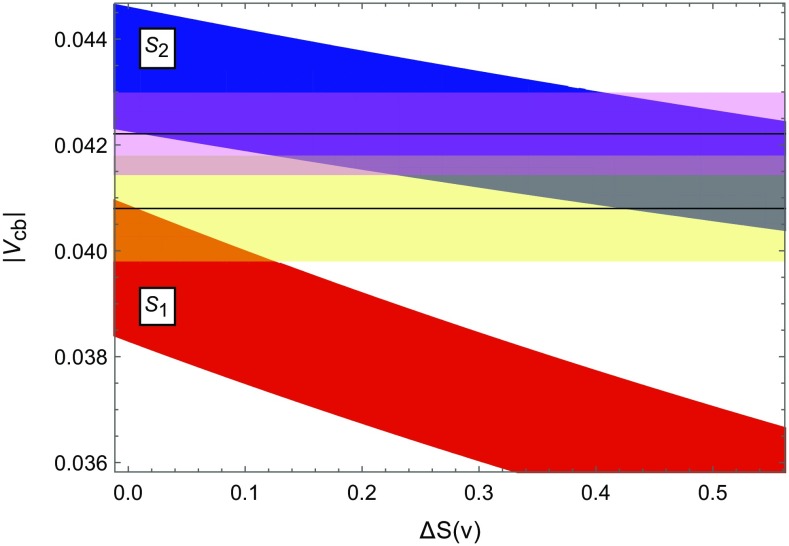
$|V_{cb}|$ versus the flavour-universal NP contribution $\Delta S(v)$ obtained in $S_1$ (*red*) and $S_2$ (*blue*). The *horizontal bands* correspond to the tree-level measurements in (22) (*yellow*) and (23) (*violet*)

This dependence is represented by the *red* band in Fig. 4 with $\Delta S(v)$ defined by

31 $$\begin{aligned} S(v)=S_0(x_t)+\Delta S(v). \end{aligned}$$

For illustrative purposes we also show the tree-level values in (22) and (23). Evidently the exclusive determinations of $|V_{cb}|$ are favoured in $S_1$. Furthermore with increasing $\Delta S(v)$, $|V_{cb}|$ quickly drops significantly below the value in (22).

Having the full CKM matrix as a function of *S*(*v*), we can calculate the CP-violating parameter $\varepsilon _K$. We use the usual formulae, which can be found in [34]. It should be noted that $\varepsilon _K$ depends directly on

32 $$\begin{aligned} V_{ts}=-|V_{ts}|\, e^{-i\beta _s},\quad V_{td}=|V_{td}|\, e^{-i\beta } \end{aligned}$$

with $\beta _s=-1^\circ$. Consequently, the value of $|V_{cb}|$ is not needed for this evaluation.

Now, the dominant contribution to $\varepsilon _K$ is proportional to

33 $$\begin{aligned} |\varepsilon _K|\propto |V_{ts}|^2|V_{td}|^2 S(v) \propto \frac{1}{S(v)}, \end{aligned}$$

where we have used (28). Thus with $|V_{ts}|$ and $|V_{td}|$ determined through $\Delta M_{s,d}$, the parameter $\varepsilon _K$ *decreases* with increasing *S*(*v*), in contrast to the analysis in which the CKM parameters are taken from tree-level decays. In that case $\varepsilon _K$ increases with increasing *S*(*v*).

Consequently using $S_1$ we find the upper bound on $\varepsilon _K$ in CMFV models to be

34 $$\begin{aligned} |\varepsilon _K|\le (1.61\pm 0.25)\cdot 10^{-3}. \end{aligned}$$

We conclude that the imposition of the $\Delta M_{s,d}$ constraints within CMFV models implies an upper bound on $\varepsilon _K$, saturated in the SM, which is significantly below its experimental value given in Table 1. Therefore a non-CMFV contribution

35 $$\begin{aligned} |\varepsilon _K|_\text {non-CMFV} \ge (0.62\pm 0.25)\cdot 10^{-3} \end{aligned}$$

is required, implying a discrepancy of the SM and CMFV value of $\varepsilon _K$ with the data by $2.5\,\sigma$. Once more we stress that this shift cannot be obtained within CMFV models without violating the constraints from $\Delta M_{s,d}$.

In Table 2 we collect the values of the most relevant CKM parameters as well as the real and imaginary parts of $\lambda _t=V_{td} V^*_{ts}$. In particular the value of $\mathrm{Im}\lambda _t$ is important for the ratio $\varepsilon '/\varepsilon$. Its value found in $S_1$ is lower than what has been used in the recent papers [50–53], thereby further decreasing the value of $\varepsilon '/\varepsilon$ in the SM.

### $S_2$: lower bounds on $\Delta M_{s,d}$

The strategy $S_2$ uses the construction of the UUT as outlined above, but then instead of using $\Delta M_s$ for the complete extraction of the CKM elements, the experimental value of $\varepsilon _K$ is used as input. Taking the lower bound in (8) into account, this strategy again implies *upper bounds* on $|V_{ts}|$, $|V_{td}|$, $|V_{cb}|$ and $|V_{ub}|$. However, this time their *S*(*v*) dependence differs from the one in (28), as seen in the case of $|V_{cb}|$ in Fig. 4, where $S_2$ is represented by the *blue* band. The weaker *S*(*v*) dependence in $S_2$, together with the higher $|V_{cb}|$ values, is another proof that the tension between $\varepsilon _K$ and $\Delta M_{s,d}$ cannot be removed within the CMFV framework and is in fact smallest in the SM limit.

In order to understand this weaker dependence of $|V_{cb}|$ on *S*(*v*) we use the formula for $|V_{cb}|$ extracted from $\varepsilon _K$, which has been derived in [34]. We recall it here for convenience^1^

36 $$\begin{aligned} |V_{cb}|= & {} \frac{\tilde{v}(\eta _{cc},\eta _{ct})}{\sqrt{\xi S(v)}}\sqrt{\sqrt{1+h(\eta _{cc},\eta _{ct}) S(v)}-1}\nonumber \\&\approx \frac{\tilde{v}(\eta _{cc},\eta _{ct})}{\sqrt{\xi }}\left[ \frac{h(\eta _{cc},\eta _{ct})}{S(v)}\right] ^{1/4}, \end{aligned}$$

where for the central values of the QCD corrections $\eta _{cc}$ and $\eta _{ct}$ in Table 1 one finds

37 $$\begin{aligned} \tilde{v}(\eta _{cc},\eta _{ct}) = 0.0282,\quad h(\eta _{cc},\eta _{ct})=24.83~. \end{aligned}$$

Values of $\tilde{v}(\eta _{cc},\eta _{ct})$ and $h(\eta _{cc},\eta _{ct})$ in the full range of $\eta _{cc}$ and $\eta _{ct}$ can be found in Table 3 of [34].

Inserting (36) into (14) we find

38 $$\begin{aligned} |V_{ts}|\propto \frac{1}{S(v)^{1/4}}, \quad |V_{td}|\propto \frac{1}{S(v)^{1/4}} \end{aligned}$$

and consequently from (9) and (10)

39 $$\begin{aligned} \Delta M_s \propto \sqrt{S(v)}, \quad \Delta M_d \propto \sqrt{S(v)}. \end{aligned}$$

Therefore, with (8), we find *lower bounds* on $\Delta M_s$ and $\Delta M_d$ that are significantly larger than the data,

40 $$\begin{aligned} \Delta M_s \ge (21.4\pm 1.8)\text {ps}^{-1}, \quad \Delta M_d \ge (0.608\pm 0.062) \text {ps}^{-1}.\nonumber \\ \end{aligned}$$

Consequently, our results for $\Delta M_s$ and $\Delta M_d$ in the SM differ from their experimental values by $2.0\sigma$ and $1.7\sigma$, respectively. This difference increases for other CMFV models. On the other hand, as seen in Fig. 4, the value of $|V_{cb}|$ in $S_2$ is fully compatible with its tree-level determination from inclusive decays, but for small $\Delta S(v)$ larger than its exclusive determination.

The ratio of the central values of $\Delta M_{s,d}$ obtained by us

41 $$\begin{aligned} \left( \frac{\Delta M_s}{\Delta M_d}\right) ^\mathrm{CMFV}= 35.1 \end{aligned}$$

perfectly agrees with the data, as this ratio is used in $S_1$ and $S_2$ as experimental input in our analysis. The error on this ratio calculated directly from (40) is spurious as we impose this ratio from experiment and the true error is negligible. Only when one individually calculates $\Delta M_s$ and $\Delta M_d$ with $|V_{cb}|$ extracted from $\varepsilon _K$, the errors in (40) are found. However, they are correlated and cancel in the ratio.

On the other hand, using the tree-level determination of the CKM matrix, the authors of [3] find in the SM

42 $$\begin{aligned} (\Delta M_s)^\mathrm{SM}&= (19.8\pm 1.5 )\,\text {ps}^{-1}, \nonumber \\ (\Delta M_d)^\mathrm{SM}&= (0.639\pm 0.063 )\,\text {ps}^{-1} \end{aligned}$$

and

43 $$\begin{aligned} \left( \frac{\Delta M_s}{\Delta M_d}\right) ^\mathrm{SM}= 31.0\pm 1.2. \end{aligned}$$

Compared with (41), this shows the inconsistency between the tree-level determination of the CKM matrix and $\Delta F=2$ processes in CMFV models.

In Table 2 we compare the results for the CKM elements obtained in $S_2$ with the ones found using $S_1$. In both cases we use the SM value for *S*(*v*), as it allows to obtain values of $\varepsilon _K$ in $S_1$ and of $\Delta M_{s,d}$ in $S_2$ closest to the data. But as we can see, the values of the CKM elements obtained in $S_2$ differ by much from the corresponding ones in $S_1$, and in particular favour the inclusive determination of $|V_{cb}|$. Also the value of $\mathrm{Im}\lambda _t$ is larger, however, it differs only by a few percent from the one used in recent calculations of $\varepsilon '/\varepsilon$ [50–53].

**Fig. 5:**
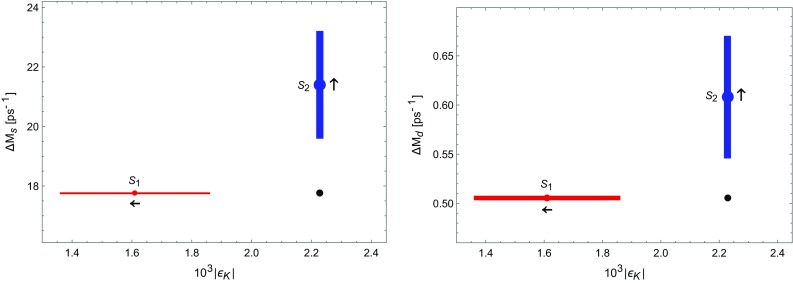
$\Delta M_{s,d}$ and $\varepsilon _K$ obtained from the strategies $S_1$ and $S_2$ for $S(v)=S_0(x_t)$, at which the upper bound on $\varepsilon _K$ in $S_1$ and lower bound on $\Delta M_{s,d}$ in $S_2$ are obtained. The *arrows* show how the *red* and *blue* regions move with increasing *S*(*v*). The *black dot* represents the experimental values

**Table 3:** CMFV predictions for various quantities as functions of *S*(*v*) and $\gamma$. The four elements of the CKM matrix are in units of $10^{-3}$, $F_{B_s} \sqrt{\hat{B}_{B_s}}$ and $F_{B_d} \sqrt{\hat{B}_{B_d}}$ in MeV and $\mathcal {B}(B^+\rightarrow \tau ^+\nu )$ in units of $10^{-4}$. From [34]

*S*(*v*)	$\gamma$	$|V_{cb}|$	$|V_{ub}|$	$|V_{td}|$	$|V_{ts}|$	$F_{B_s}\sqrt{\hat{B}_{B_s}}$	$F_{B_d} \sqrt{\hat{B}_{B_d}}$	$\xi$	$\mathcal {B}(B^+\rightarrow \tau ^+\nu )$
2.31	$63^\circ$	43.6	3.69	8.79	42.8	252.7	210.0	1.204	0.822
2.5	$63^\circ$	42.8	3.63	8.64	42.1	247.1	205.3	1.204	0.794
2.7	$63^\circ$	42.1	3.56	8.49	41.4	241.8	200.9	1.204	0.768

We conclude therefore, as already indicated by the analysis in [34], that it is impossible within CMFV models to obtain a simultaneous agreement of $\Delta M_{s,d}$ and $\varepsilon _K$ with the data. The improved lattice results in (3) and (4) allow one to exhibit this difficulty stronger. In the context of the strategies $S_1$ and $S_2$, the tension between $\Delta M_{d,s}$ and $\varepsilon _K$ is summarised by the plots of $\Delta M_{s,d}$ vs. $\varepsilon _K$ in Fig. 5. Note that these plots differ from the known plots of $\Delta M_{s,d}$ vs. $\varepsilon _K$ in CMFV models (see e.g. Fig. 5 in [2]). In the latter plot the CKM parameters were taken from tree-level decays, and varying *S*(*v*) increased both $\Delta M_{s,d}$ and $\varepsilon _K$ in a correlated manner. Even if the physics in those plots and in the plots in Fig. 5 is the same, presently the accuracy of the outcome of strategies $S_1$ and $S_2$ shown in Fig. 5 is higher.

The problems with CMFV models encountered here could be anticipated on the basis of the first three rows of Table 2 from [34], which we recall in Table 3. In that paper a different strategy has been used and various quantities have been predicted in CMFV models as functions of *S*(*v*) and $\gamma$. As the first three columns correspond to $\gamma =63^\circ$ and $\xi =1.204$, very close to the values of these quantities found in the present paper, there is a clear message from Table 3. The predicted values of $F_{B_s}\sqrt{\hat{B}_{B_s}}$ and $F_{B_d} \sqrt{\hat{B}_{B_d}}$ are significantly below their recent values from [3] in (3). Moreover, with increasing *S*(*v*) there is a clear disagreement between the values of these parameters favoured by CMFV and the values in (3). We also refer to the plots in Fig. 4 of [34], where the correlations between $|V_{cb}|$ and $F_{B_d} \sqrt{\hat{B}_{B_d}}$ and between $|V_{cb}|$ and $F_{B_s} \sqrt{\hat{B}_{B_s}}$ implied by CMFV have been shown. Already in 2013 there was some tension between the grey regions in that figure representing the 2013 lattice values and the CMFV predictions. With the 2016 lattice values in (3), the grey areas shrunk and moved away from the values favoured by CMFV. Other problems of CMFV seen from the point of view of the strategy in [34] are listed in Sect. 3 of that paper.

## Implications for rare *K* and *B* decays in the SM

In the previous section we have determined the full CKM matrix using in turn the strategies $S_1$ and $S_2$. It is interesting to determine the impact of these new determinations on the branching ratios of the rare decays $K^+\rightarrow \pi ^+\nu \bar{\nu }$, $K_{L}\rightarrow \pi ^0\nu \bar{\nu }$ and $B_{s,d}\rightarrow \mu ^+\mu ^-$ within the SM. To this end we use for $K^+\rightarrow \pi ^+\nu \bar{\nu }$ and $K_{L}\rightarrow \pi ^0\nu \bar{\nu }$ the parametric formulae derived in [54] which we recall here for completeness:

44 $$\begin{aligned} \mathcal {B}(K^+\rightarrow \pi ^+\nu \bar{\nu })_\text {SM}= & {} (8.39 \pm 0.30) \cdot 10^{-11} \nonumber \\&\times \left[ \frac{\left| V_{cb}\right| }{40.7\cdot 10^{-3}}\right] ^{2.8}\left[ \frac{\gamma }{73.2^\circ }\right] ^{0.74},\nonumber \\ \end{aligned}$$

45 $$\begin{aligned} \mathcal {B}(K_{L}\rightarrow \pi ^0\nu \bar{\nu })_\text {SM}= & {} (3.36 \pm 0.05) \cdot 10^{-11} \nonumber \\&\times \left[ \frac{\left| V_{ub}\right| }{3.88\cdot 10^{-3}}\right] ^2\left[ \frac{\left| V_{cb}\right| }{40.7\cdot 10^{-3}}\right] ^2\nonumber \\&\times \left[ \frac{\sin (\gamma )}{\sin (73.2^\circ )}\right] ^{2}. \end{aligned}$$

For $B_{s}\rightarrow \mu ^+\mu ^-$ we use the formula from [55], slightly modified in [2]

46 $$\begin{aligned} \overline{\mathcal {B}}(B_{s}\rightarrow \mu ^+\mu ^-)_\mathrm{SM}= & {} (3.65\pm 0.06)\cdot 10^{-9} \nonumber \\&\times \left[ \frac{m_t(m_t)}{163.5 \, \mathrm{GeV}}\right] ^{3.02}\nonumber \\&\times \left[ \frac{\alpha _s(M_Z)}{0.1184}\right] ^{0.032} R_s \end{aligned}$$

**Table 4:** SM predictions for rare decay branching ratios using the strategies $S_1$ and $S_2$, as explained in the text

$S_i$	${\mathcal {B}}(K^+\rightarrow \pi ^+\nu \bar{\nu })$	${\mathcal {B}}(K_{L}\rightarrow \pi ^0\nu \bar{\nu })$	$\overline{\mathcal {B}}(B_{s}\rightarrow \mu ^+\mu ^-)$	$\mathcal {B}(B_{d}\rightarrow \mu ^+\mu ^-)$
$S_1$	$6.88\,(70)\cdot 10^{-11}$	$2.11\,(25)\cdot 10^{-11}$	$3.14\,(22)\cdot 10^{-9}$	$0.84\,(7)\cdot 10^{-10}$
$S_2$	$8.96\,(79)\cdot 10^{-11}$	$3.08\,(32)\cdot 10^{-11}$	$3.78\,(23)\cdot 10^{-9}$	$1.02\,(8)\cdot 10^{-10}$

where

47 $$\begin{aligned} R_s= & {} \left[ \frac{F_{B_s}}{227.7\, \mathrm{MeV}}\right] ^2 \left[ \frac{\tau _{B_s}}{1.516 \mathrm{ps}}\right] \left[ \frac{0.938}{r(y_s)}\right] \nonumber \\&\times \left[ \frac{|V_{ts}|}{41.5\cdot 10^{-3}}\right] ^2. \end{aligned}$$

The “bar” in (46) indicates that $\Delta \Gamma _s$ effects [56–58] have been taken into account through

48 $$\begin{aligned} r(y_s)=1-y_s, \quad y_s\equiv \tau _{B_s}\frac{\Delta \Gamma _s}{2} =0.062\pm 0.005. \end{aligned}$$

For $B_d\rightarrow \mu ^+\mu ^-$ one finds [55]

49 $$\begin{aligned}&\mathcal {B}(B_{d}\rightarrow \mu ^+\mu ^-)_\mathrm{SM} = (1.06\pm 0.02)\cdot 10^{-10} \nonumber \\&\quad \times \left[ \frac{m_t(m_t)}{163.5 \, \mathrm{GeV}}\right] ^{3.02}\left[ \frac{\alpha _s(M_Z)}{0.1184}\right] ^{0.032} R_d \end{aligned}$$

where

50 $$\begin{aligned} R_d=\left[ \frac{F_{B_d}}{190.5\, \mathrm{MeV}}\right] ^2 \left[ \frac{\tau _{B_d}}{1.519 \mathrm{ps}}\right] \left[ \frac{|V_{td}|}{8.8\cdot 10^{-3}}\right] ^2. \end{aligned}$$

In Table 4 we collect the results for the four branching ratios in the SM obtained using the strategies $S_1$ and $S_2$ for the determination of the CKM parameters and other updated parameters collected in Table 1. We observe significant differences in these two determinations, which gives another support for the tension between $\Delta M_{s,d}$ and $\varepsilon _K$ in the SM, holding more generally in CMFV models.

Our results for $B_{s,d}\rightarrow \mu ^+\mu ^-$ should be compared with the results of the combined analysis of CMS and LHCb data [59]

51 $$\begin{aligned} \overline{\mathcal {B}}(B_{s}\rightarrow \mu ^+\mu ^-)= & {} (2.8^{+0.7}_{-0.6}) \cdot 10^{-9},\nonumber \\ \mathcal {B}(B_{d}\rightarrow \mu ^+\mu ^-)= & {} (3.9^{+1.6}_{-1.4})\cdot 10^{-10}. \end{aligned}$$

We observe that in $S_1$ the SM prediction for $B_s\rightarrow \mu ^+\mu ^-$ is rather close to the data, while in the case of $S_2$ it is visibly larger.

Finally, in view of the improved lattice determinations of the parameters $\hat{B}_{B_s}$ and $\hat{B}_{B_d}$ [3]

52 $$\begin{aligned} \hat{B}_{B_s}=1.49\pm 0.09,\quad \hat{B}_{B_d}=1.49\pm 0.13 \end{aligned}$$

it is tempting to calculate the $B_{s,d}\rightarrow \mu ^+\mu$ branching ratios by normalizing them to $\Delta M_{s,d}$ [60]. This eliminates not only the dependence on the CKM parameters and weak decay constants, but also reduces the dependence on $m_t$. Neglecting the tiny uncertainties in $\eta _B$, $\alpha _s$ and $\tau _{B_q}$ we find the very accurate expressions

53 $$\begin{aligned}&\overline{\mathcal {B}}(B_{s}\rightarrow \mu ^+\mu ^-)_\mathrm{SM} = (3.14\pm 0.05)\cdot 10^{-9} \nonumber \\&\quad \times \left[ \frac{1.49}{\hat{B}_{B_s}}\right] \left[ \frac{0.938}{r(y_s)}\right] \left[ \frac{m_t(m_t)}{163.5 \, \mathrm{GeV}}\right] ^{1.5},\end{aligned}$$

54 $$\begin{aligned}&{\mathcal {B}}(B_{d}\rightarrow \mu ^+\mu ^-)_\mathrm{SM} = (0.84\pm 0.02)\cdot 10^{-10} \nonumber \\&\quad \times \left[ \frac{1.49}{\hat{B}_{B_d}}\right] \left[ \frac{m_t(m_t)}{163.5 \, \mathrm{GeV}}\right] ^{1.5}. \end{aligned}$$

These expressions apply only to the SM and $S_1$, where the experimental values of $\Delta M_{s,d}$ are used to determine the CKM matrix. We then find

55 $$\begin{aligned} \overline{\mathcal {B}}(B_{s}\rightarrow \mu ^+\mu ^-)_\text {SM}= & {} (3.14\pm 0.19) \cdot 10^{-9},\nonumber \\ \mathcal {B}(B_{d}\rightarrow \mu ^+\mu ^-)_\text {SM}= & {} (0.84\pm 0.07)\cdot 10^{-10}. \end{aligned}$$

These results agree perfectly with the ones in Table 4. This is not surprising because in $S_1$ the constraint from $\Delta M_{s,d}$ has been imposed and the authors of [3] extracted the values of $\hat{B}_{B_q}$ from their results in (3) and $F_{B_q}$ in Table 1. The outcome of this exercise will be more illuminating once independent and more precise lattice determinations of the $\hat{B}_{B_{s,d}}$ parameters become available. In addition, the derived formulae (53) and (54) are much simpler than the ones in (46) and (49), respectively. They allow in no time to calculate the branching ratios in question in terms of $\hat{B}_{B_s}$, $\hat{B}_{B_d}$, $\Delta \Gamma _s$ and $m_t$.

## Beyond CMFV

Our analysis of CMFV models signals the violation of flavour universality in the function *S*(*v*), signalling the presence of new sources of flavour and CP-violation and/or new operators contributing to $\Delta F=2$ transitions beyond the SM $(V-A)\otimes (V-A)$ ones.^2^ For simplicity we will here restrict ourselves to solutions in which only SM operators are present.

A fully general and very convenient solution in this case is just to consider instead of the flavour-universal function *S*(*v*) three functions

56 $$\begin{aligned} S_i=|S_i| e^{i\varphi _i}, \quad i=K,s,d. \end{aligned}$$

It is evident that with two free parameters in each meson system it is possible to obtain agreement with the data on $\Delta F=2$ observables. The simplest models of this type are models with tree-level $Z^\prime$ and *Z* exchanges analysed in detail in [62]. The flavour violating couplings in these models are complex numbers (two free parameters) and can be chosen in such a manner that any problems of CMFV models in $\Delta F=2$ processes are removed by properly choosing these couplings. Effectively the observables in (1) are simply used to find these parameters or equivalently $S_i$. The test of these scenarios is only offered through the correlations with $\Delta F=1$ processes, that is rare *K* or $B_{s,d}$ decays, which in these simple models involve the same couplings. The analysis in [62] then shows that when constraints from $\Delta F=1$ processes are taken into account it is easier to obtain agreement with the data for $\Delta F=2$ processes in the case of $Z^\prime$ models than models with tree-level *Z* exchanges.

Here we would like to discuss only the models with a minimally broken $U(2)^3$ flavour symmetry [63, 64] which are more constrained. In these models, as discussed in detail in [65], in addition to the unitary CKM matrix one has

57 $$\begin{aligned} S_K=r_K S_0(x_t),\quad r_K\ge 1 \end{aligned}$$

and

58 $$\begin{aligned} |S_d|=|S_s|=r_B S_0(x_t), \quad \varphi _{d}=\varphi _{s}\equiv \varphi _\mathrm{new} \end{aligned}$$

with $r_B$ being a real parameter which could be larger or smaller than unity. The important difference from the CMFV scenario is that it cannot be tested without invoking tree-level determinations of at least some elements of the CKM matrix. The main features of this scenario are:

- No correlation between the *K* and $B_{s,d}$ systems, so that the tension between $\varepsilon _K$ and $\Delta M_{s,d}$ is absent in these models.
- However, as $r_K\ge 1$, finding one day $\varepsilon _K$ in the SM to be larger than the data would exclude this scenario. Presently such a situation seems rather unlikely.
- $S_d \equiv S_s$ are complex functions and $r_B$ can be larger or smaller than unity. Consequently, through interference with the SM contributions, $\Delta M_{s,d}$ can be suppressed or enhanced as needed.
- With the new phase $\varphi _\mathrm{new}$ and $r_B$ not bounded from below there is more freedom than in the CMFV scenario.

However, due to the equality $S_d=S_s$ there are two important implications that can be tested.

The first one is the CMFV relation [65]

59 $$\begin{aligned} \left( \frac{\Delta M_d}{\Delta M_s}\right) _{\mathrm{MU}(2)^3}= & {} \left( \frac{\Delta M_d}{\Delta M_s}\right) _\mathrm{CMFV}= \left( \frac{\Delta M_d}{\Delta M_s}\right) _\mathrm{SM}\nonumber \\= & {} \frac{m_{B_d}}{m_{B_s}} \frac{1}{\xi ^2} \left| \frac{V_{td}}{V_{ts}}\right| ^2. \end{aligned}$$

from which one can obtain the ratio $|V_{td}|/|V_{ts}|$ as done already in Sect. 2, see (12), which can be compared with its tree-level determination. As stated before, the tree-level determination of this ratio, quoted in (13), is significantly larger, and consequently M$U(2)^3$ models have the same difficulty here as CMFV models. Yet, a firm conclusion will only be reached after the result in (13) will be superseded by a more precise tree-level determination of the angle $\gamma$.

The second one is the correlation between the two CP-asymmetries that results from the equality of NP phases in

60 $$\begin{aligned}&S_{\psi K_S} = \sin (2\beta +2\varphi _\mathrm{new}), \nonumber \\&S_{\psi \phi } = \sin (2|\beta _s|-2\varphi _\mathrm{new}),\quad (\mathrm{M}U(2)^3). \end{aligned}$$

As $\beta _s$ is very small in the SM, a precise measurement of $S_{\psi \phi }$ determines $\varphi _\mathrm{new}$. From the measured value of $S_{\psi K_S}$ we then obtain $\beta$. The latter value can be compared with the one obtained from the tree-level determination of $|V_{ub}|/|V_{cb}|$ and either $R_t$ or the tree-level determination of $\gamma$. However, $\beta$ is strongly correlated with $|V_{ub}|/|V_{cb}|$, with very weak dependence on $\gamma$ and $R_t$. Therefore eventually (60) implies a triple correlation between [65]

61 $$\begin{aligned} S_{\psi K_S},\quad S_{\psi \phi },\quad \frac{|V_{ub}|}{|V_{cb}|}, \end{aligned}$$

which provides another important test of the M$U(2)^3$ scenario once the three observables will be known precisely.

In summary, M$U(2)^3$ models match the new lattice data better than CMFV, but similar to the latter models they have difficulties with the value of $\gamma$ and of the ratio $|V_{td}|/|V_{ts}|$ being significantly below their tree-level determinations.

Concerning more complicated models like the Littlest Higgs model with T-parity [66, 67] or 331 models [68], it is clear that the new lattice data have an impact on the allowed ranges of new parameters. However, such a study is beyond the scope of our paper.

## Conclusions

In this paper we have determined the Universal Unitarity Triangle (UUT) of constrained minimal flavour violation (CMFV) models. We then derived the full CKM matrix, using either the experimental value of $\Delta M_s$ or of $|\varepsilon _K|$ as input. The recently improved values of the hadronic matrix elements in (3) and (4) [3] have been crucial for this study. In contrast to many analyses in the literature, we avoided tree-level determinations of $|V_{ub}|$ and $|V_{cb}|$.

The main messages from this analysis are as follows:

- The extracted angle $\gamma$ in the UUT is already known precisely and is significantly smaller than its tree-level determination. This is a direct consequence of the small value of $\xi$ in (4). In turn the ratio $|V_{td}|/|V_{ts}|$ also turns out to be smaller than its tree-level determination, as already pointed out in [3].
- The precise relation between $|V_{ub}|$ and $|V_{cb}|$ obtained by us in (21) provides another test of CMFV. See Fig. 2.
- Requiring CMFV to reproduce the data for $\Delta M_{s,d}$ (strategy $S_1$), we find that low values of $|V_{ub}|$ and $|V_{cb}|$ are favoured, in agreement with their exclusive determinations. More importantly we derived an upper bound on $|\varepsilon _K|$ that is significantly below the data.
- Requiring CMFV to reproduce the data for $\varepsilon _K$ (strategy $S_2$), we find a higher value of $|V_{ub}|$, still consistent with exclusive determinations, but $|V_{cb}|$ significantly higher than in $S_1$ and in agreement with its inclusive determination. The derived lower bounds on $\Delta M_{s,d}$ are then significantly above the data.
- The tension between $\varepsilon _K$ and $\Delta M_{s,d}$ in CMFV models with either $|\varepsilon _K|$ being too small or $\Delta M_{s,d}$ being too large cannot be removed by varying *S*(*v*). This would only be possible, as stressed in [34], if the values in (3) turned out to be significantly smaller and $\xi$ larger than in (4). With the present values of these parameters, the SM performs best among all CMFV models, even if, as seen in Fig. 5, it falls short in properly describing the $\Delta F=2$ data.
- The inconsistency of $\Delta M_{d,s}$ and $\varepsilon _K$ in the SM and CMFV is also signalled by rather different predictions for rare decay branching ratios obtained using strategies $S_1$ and $S_2$. See Sect. 3 and Table 4.
- As the correlation between $\varepsilon _K$ and $\Delta M_{s,d}$ is broken in models with $U(2)^3$ flavour symmetry, these models perform better than CMFV models. Still the correlation between $\Delta M_s$ and $\Delta M_d$, that is of CMFV type, predicted by these models is in conflict with the tree-level determinations already pointed out in [3] within the SM. See (12) and (13).

Our analysis of CMFV models shows that they fail to properly describe the existing data on $\Delta F=2$ observables simultaneously and implies thereby the presence of either new sources of flavour violation and/or new operators. Several models analysed in the literature like $Z^\prime$ models, 331 models, or the Littlest Higgs model with T-parity could help in bringing the theory to agree with the data. Firm conclusions would, however, require a dedicated study.

Certainly, further improvements on the hadronic matrix elements from lattice QCD and on the tree-level determinations of $|V_{ub}|$, $|V_{cb}|$ and $\gamma$ will sharpen the prediction for the size of required NP contributions to $\Delta F=2$ observables, thereby selecting models which could bring the theory to agree with experimental data. In particular finding the value of $\gamma$ from tree-level determinations in the ballpark of $70^\circ$ would imply the violation of the CMFV relation (59). On the other hand, resolving the discrepancy between exclusive and inclusive tree-level determinations of $|V_{ub}|$ in favour of the latter, would indicate the presence of new CP-violating phases affecting $S_{\psi K_S}$. Moreover, the correlations of $\Delta F=2$ transitions with rare *K* and $B_{s,d}$ decays and $\varepsilon '/\varepsilon$ could eventually give us a deeper insight into the NP at short distance scales that is responsible for the anomalies indicated by the new lattice data, as reviewed in [2] and recently stressed in [53].

## Footnotes

1 We replaced $v(\eta _{cc},\eta _{ct})$ by $\tilde{v}(\eta _{cc},\eta _{ct})$ in order to distinguish it from the argument in *S*(*v*).

2 In a more general formulation of MFV new operators could be present [61].

## References

[1] Isidori G, Nir Y, Perez G (2010). Flavor physics constraints for physics beyond the standard model. Ann. Rev. Nucl. Part. Sci..

[2] Buras AJ, Girrbach J (2014). Towards the identification of new physics through quark flavour violating processes. Rept. Prog. Phys..

[3] A. Bazavov et al., $$B^0_{(s)}$$-mixing matrix elements from lattice QCD for the Standard Model and beyond. arXiv:1602.03560

[4] Aoki S, Aoki Y, Bernard C, Blum T, Colangelo G (2014). Review of lattice results concerning low-energy particle physics. Eur. Phys. J. C.

[5] ETM collaboration, N. Carrasco et al., $$B$$ tmQCD: the Standard Model and beyond. JHEP **03**, 016 (2014). arXiv:1308.1851

[6] Aoki Y, Arthur R, Blum T, Boyle P, Brommel D (2011). Continuum limit of $$B_K$$ from 2+1 flavor domain wall QCD. Phys. Rev. D.

[7] Bae T, Jang Y-C, Jung C, Kim H-J, Kim J (2010). $$B_K$$ using HYP-smeared staggered fermions in $$N_f=2+1$$ unquenched QCD. Phys. Rev. D.

[8] ETM collaboration, M. Constantinou et al., $$B_K$$ = 2 *twisted mass lattice QCD*, *Phys. Rev. D* **83**, 014505 (2011). arXiv:1009.5606

[9] Colangelo G, Durr S, Juttner A, Lellouch L, Leutwyler H (2011). Review of lattice results concerning low energy particle physics. Eur. Phys. J. C.

[10] J.A. Bailey, T. Bae, Y.-C. Jang, H. Jeong, C. Jung, et al., Beyond the standard model corrections to $$K^0-\bar{K}^0$$ mixing. PoS LATTICE **2012**, 107 (2012). arXiv:1211.1101

[11] Durr S, Fodor Z, Hoelbling C, Katz S, Krieg S (2011). Precision computation of the kaon bag parameter. Phys. Lett. B.

[12] A. Vladikas, FLAG: Lattice QCD tests of the standard model and foretaste for beyond. PoS FPCP **2015**, 016 (2015). arXiv:1509.01155

[13] Gaiser BD, Tsao T, Wise MB (1981). Parameters of the six quark model. Ann. Phys..

[14] Buras AJ, Gérard J-M (1986). $$1/N$$ expansion for kaons. Nucl. Phys. B.

[15] J.-M. Gérard, An upper bound on the Kaon B-parameter and $${\rm Re}(\epsilon _K)$$. JHEP **1102**, 075 (2011). arXiv:1012.2026

[16] Buras AJ, Gérard J-M, Bardeen WA (2014). Large $$N$$ approach to kaon decays and mixing 28 years later: $$\Delta I = 1/2$$ rule, $$\hat{B}_K$$ and $$\Delta M_K$$. Eur. Phys. J. C.

[17] Buras AJ, Guadagnoli D, Isidori G (2010). On $$\epsilon _K$$ beyond lowest order in the operator product expansion. Phys. Lett. B.

[18] Brod J, Gorbahn M (2012). Next-to-Next-to-Leading-Order Charm-Quark Contribution to the CP Violation Parameter $$\varepsilon _K$$ and $$\Delta M_K$$. Phys. Rev. Lett..

[19] CKMfitter Group collaboration, K. Trabelsi, World average and experimental overview of $$\gamma /\varphi _3$$; presented at CKM 2014

[20] Fleischer R, Knegjens R (2011). In Pursuit of New Physics with $$B^0_s\rightarrow K^+K^-$$. Eur. Phys. J. C.

[21] LHCb collaboration, R. Aaij et al., Determination of $$\gamma $$ from charmless two-body decays of beauty mesons. Phys. Lett. B **741**, 1–11 (2015). arXiv:1408.4368

[22] Goto T, Kitazawa N, Okada Y, Tanaka M (1996). Model independent analysis of $$B \bar{B}$$ mixing and CP violation in $$B$$ decays. Phys. Rev. D.

[23] Buras AJ, Gambino P, Gorbahn M, Jager S, Silvestrini L (2001). Universal unitarity triangle and physics beyond the standard model. Phys. Lett. B.

[24] Buras AJ (2003). Minimal flavor violation. Acta Phys. Polon. B.

[25] Blanke M, Buras AJ, Guadagnoli D, Tarantino C (2006). Minimal flavour violation waiting for precise measurements of $$\Delta M_s$$, $$S_{\psi \phi }$$, $$A^s_\text{ SL }$$, $$|V_{ub}|$$, $$\gamma $$ and $$B^0_{s, d} \rightarrow \mu ^+ \mu ^-$$. JHEP.

[26] Blanke M, Buras AJ (2007). Lower bounds on $$\Delta M_{s, d}$$ from constrained minimal flavour violation. JHEP.

[27] Buras AJ, Carlucci MV, Merlo L, Stamou E (2012). Phenomenology of a gauged $$SU(3)^3$$ flavour model. JHEP.

[28] Buras AJ, Girrbach J (2012). BSM models facing the recent LHCb data: a first look. Acta Phys. Polon. B.

[29] Lunghi E, Soni A (2008). Possible indications of new physics in $$B_d$$-mixing and in $$\sin (2 \beta )$$ determinations. Phys. Lett. B.

[30] Buras AJ, Guadagnoli D (2008). Correlations among new CP violating effects in $$\Delta F = 2$$ observables. Phys. Rev. D.

[31] UTfit collaboration, M. Bona et al., An improved standard model prediction of $$BR(B\rightarrow \tau \nu )$$ and its implications for new physics. Phys. Lett. B **687**, 61–69 (2010). arXiv:0908.3470

[32] Lenz A, Nierste U, Charles J, Descotes-Genon S, Jantsch A (2011). Anatomy of new physics in $$B - \bar{B}$$ mixing. Phys. Rev. D.

[33] Lunghi E, Soni A (2011). Possible evidence for the breakdown of the CKM-paradigm of CP-violation. Phys. Lett. B.

[34] A.J. Buras, J. Girrbach, Stringent tests of constrained minimal flavour violation through $$\Delta F=2$$ transitions. Eur. Phys. J. C **9**, 73 (2013). arXiv:1304.6835

[35] Buras AJ, Jamin M, Weisz PH (1990). Leading and next-to-leading QCD corrections to $$\varepsilon $$ parameter and $$B^0-\bar{B}^0$$ mixing in the presence of a heavy top quark. Nucl. Phys. B.

[36] Particle Data Group collaboration, K. Olive et al., Review of particle physics. Chin. Phys. C **38**, 090001 (2014)

[37] Heavy Flavor Averaging Group (HFAG) Collaboration, Y. Amhis et al., Averages of $$b$$-lepton properties as of summer 2014. arXiv:1412.7515

[38] J.L. Rosner, S. Stone, R.S. Van de Water, Leptonic Decays of Charged Pseudoscalar Mesons (2015). arXiv:1509.02220

[39] HPQCD collaboration, R. J. Dowdall, C. T. H. Davies, R. R. Horgan, C. J. Monahan and J. Shigemitsu, B-Meson Decay Constants from Improved Lattice Nonrelativistic QCD with Physical u, d, s, and c Quarks, *Phys. Rev. Lett.* **110**, 222003 (2013). arXiv:1302.2644 10.1103/PhysRevLett.110.222003 23767714

[40] Brod J, Gorbahn M (2010). $$\epsilon _K$$ at next-to-next-to-leading order: the charm-top-quark contribution. Phys. Rev. D.

[41] Urban J, Krauss F, Jentschura U, Soff G (1998). Next-to-leading order QCD corrections for the $$B^0 - \bar{B}^0$$ mixing with an extended Higgs sector. Nucl. Phys. B.

[42] J. Charles et al., Current status of the Standard Model CKM fit and constraints on $$\Delta F=2$$. N. Phys. Phys. Rev. D **91**, 073007 (2015). arXiv:1501.05013

[43] UTfit Collaboration, M. Bona et al., The unitarity triangle fit in the standard model and hadronic parameters from lattice QCD: a reappraisal after the measurements of $$\Delta m(s)$$. JHEP **0610**, 081 (2006). arXiv:hep-ph/0606167

[44] MILC collaboration, J.A. Bailey et al., BD form factors at nonzero recoil and $$|V_{cb}|$$ from 2+1-flavor lattice QCD. Phys. Rev. D **92**, 034506 (2015). arXiv:1503.07237

[45] Fermilab Lattice, MILC collaboration, J.A. Bailey et al., $$|V_{ub}|$$ decays and (2+1)-flavor lattice QCD. Phys. Rev. D **92**, 014024 (2015). arXiv:1503.07839

[46] Belle collaboration, R. Glattauer et al., Measurement of the decay $$B\rightarrow D\ell \nu _\ell $$. Phys. Rev. D **93**(3), 032006 (2016). arXiv:1510.03657

[47] Alberti A, Gambino P, Healey KJ, Nandi S (2015). Precision determination of the Cabibbo–Kobayashi–Maskawa element $$V_{cb}$$. Phys. Rev. Lett..

[48] LHCb Collaboration, R. Aaij et al., Determination of the quark coupling strength $$|V_{ub}|$$ using baryonic decays. Nature Phys. **11**, 743–747 (2015). arXiv:1504.01568

[49] D. Du, A.X. El-Khadra, S. Gottlieb, A.S. Kronfeld, J. Laiho, E. Lunghi et al., Phenomenology of semileptonic $$B$$-meson decays with form factors from lattice QCD. arXiv:1510.02349

[50] RBC, UKQCD collaboration, Z. Bai et al., Standard model prediction for direct CP violation in $$K$$ decay. Phys. Rev. Lett. **115**, 212001 (2015). arXiv:1505.07863 10.1103/PhysRevLett.115.212001 26636846

[51] A.J. Buras, M. Gorbahn, S. Jäger, M. Jamin, Improved anatomy of $$\varepsilon ^{\prime }/\varepsilon $$ in the Standard Model. JHEP **11**, 202 (2015). arXiv:1507.06345

[52] A.J. Buras, J.M. Gerard, Upper bounds on $$\varepsilon ^{\prime }/\varepsilon $$ from large N QCD and other news. JHEP **12**, 008 (2015). arXiv:1507.06326

[53] A.J. Buras, New physics patterns in $$\varepsilon ^\prime /\varepsilon $$. arXiv:1601.00005

[54] A.J. Buras, D. Buttazzo, J. Girrbach-Noe, R. Knegjens, $$ {K}^{+}\rightarrow {\pi }^{+}\nu \overline{\nu }$$ in the Standard Model: status and perspectives. JHEP **11**, 033 (2015). arXiv:1503.02693

[55] Bobeth C, Gorbahn M, Hermann T, Misiak M, Stamou E (2014). $$B_{s, d}\rightarrow \ell ^+ \ell ^-$$ in the standard model with reduced theoretical uncertainty. Phys. Rev. Lett..

[56] Descotes-Genon S, Matias J, Virto J (2012). An analysis of $$B_{d, s}$$ mixing angles in presence of new physics and an update of $$B_s \rightarrow K^{0*} \bar{K}^{0*}$$. Phys. Rev. D.

[57] De Bruyn K, Fleischer R, Knegjens R, Koppenburg P, Merk M (2012). Branching ratio measurements of $$B_s$$ decays. Phys. Rev. D.

[58] De Bruyn K, Fleischer R, Knegjens R, Koppenburg P, Merk M (2012). Probing new physics via the $$B^0_s\rightarrow \mu ^+\mu ^-$$ effective lifetime. Phys. Rev. Lett..

[59] LHCb, CMS Collaboration, V. Khachatryan et al., Observation of the rare $$B^0_s\rightarrow \mu ^+\mu ^-$$ decay from the combined analysis of CMS and LHCb data. Nature **522**, 68–72 (2015). arXiv:1411.4413 10.1038/nature14474 26047778

[60] A.J. Buras, Relations between $$\Delta M_{s, d}$$ and $$B_{s, d} \rightarrow \mu ^+ \mu ^-$$ in models with minimal flavour violation. Phys. Lett. B **566**, 115–119 (2003). arXiv:hep-ph/0303060

[61] D’Ambrosio G, Giudice GF, Isidori G, Strumia A (2002). Minimal flavour violation: an effective field theory approach. Nucl. Phys. B.

[62] Buras AJ, De Fazio F, Girrbach J (2013). The anatomy of Z’ and Z with flavour changing neutral currents in the flavour precision era. JHEP.

[63] Barbieri R, Isidori G, Jones-Perez J, Lodone P, Straub DM (2011). U(2) and minimal flavour violation in supersymmetry. Eur. Phys. J. C.

[64] Barbieri R, Buttazzo D, Sala F, Straub DM (2012). Flavour physics from an approximate $$U(2)^3$$ symmetry. JHEP.

[65] Buras AJ, Girrbach J (2013). On the correlations between flavour observables in minimal $$U(2)^3$$ models. JHEP.

[66] Blanke M (2006). Particle antiparticle mixing, $$\varepsilon _K$$, $$\Delta \Gamma _q$$, $$A_\text{ SL }^q$$, $$A_\text{ CP }(B_d \rightarrow \psi K_S)$$, $$A_\text{ CP }(B_s \rightarrow \psi \phi )$$ and $$B \rightarrow X_{s, d} \gamma $$ in the Littlest Higgs model with T- parity. JHEP.

[67] M. Blanke, A.J. Buras, S. Recksiegel, Quark flavour observables in the Littlest Higgs model with T-parity after LHC Run 1. Eur. Phys. J. C **76**(4), 182 (2016). arXiv:1507.06316 10.1140/epjc/s10052-016-4019-7 PMC5312162 28260968

[68] A.J. Buras, F. De Fazio, $$\varepsilon ^{\prime }/\varepsilon $$ in 331 models. JHEP **1603**, 010 (2016) arXiv:1512.02869

